# Apical Fracture of One‐Piece Titanium–Zirconium Mini‐Implant for Mandibular Overdenture: A Prospective 3‐Year Follow‐Up

**DOI:** 10.1111/cid.70084

**Published:** 2025-08-25

**Authors:** Cláudio Rodrigues Leles, Thalita Fernandes Fleury Curado, José Luiz Rodrigues Leles, Nádia Lago Costa, Manrique Fonseca, Martin Schimmel, Gerald McKenna

**Affiliations:** ^1^ School of Dentistry Federal University of Goias Goiania Goias Brazil; ^2^ Department of Reconstructive Dentistry and Gerodontology School of Dental Medicine, University of Bern Bern Switzerland; ^3^ Clinical Practice Goiania Brazil; ^4^ Division of Gerodontology and Removable Prosthodontics University Clinics of Dental Medicine, University of Geneva Geneva Switzerland; ^5^ Centre for Public Health Queen's University Belfast Belfast UK; ^6^ Clinic of Special Care and Geriatric Dentistry University of Zurich Zürich Switzerland

**Keywords:** case report, dental implant, implant fracture, mini‐implant, overdenture treatment

## Abstract

**Objectives:**

To assess the 3‐year clinic‐radiographic features and risk factors for apical fracture of mini‐implants for mandibular overdentures.

**Material and Methods:**

Participants in a clinical trial (*n* = 74) were assessed for post‐insertion identification of apical fractures of one‐piece titanium–zirconium mini‐implants (Straumann Mini‐Implant System). Fractures were identified during implant insertion or in follow‐up imaging exams. Cases were clinically and radiographically monitored up to the 3‐year follow‐up.

**Results:**

Five implants in five patients had apical fractures (1.69% of the 296 inserted implants). No clinical signs of peri‐implant complications were found, and overall treatment outcomes were positive for all patients. Satisfactory osseointegration was achieved, and no evidence of periapical pathology was noted on panoramic radiographs. The patients were notified about the apical implant fracture at the time of the surgery, and during follow‐up appointments, they were reassured about the absence of further complications in the long term. It was hypothesized that the increased risk of fracture was associated with high insertion torques (around 80 N cm) and implant insertion in areas where the tip engages into a hard basal cortical bone (Types I–II) with an inclined cortical surface in the lingual aspect of the alveolar bone.

**Conclusions:**

The incidence of apical fracture was low and did not result in any clinically relevant complications and may be detected during the surgery or in follow‐up radiographs. Although the intraosseous implant tip fragment may not be of major concern, additional caution to prevent excessive insertion torque and to avoid anatomical regions of higher risk may be advisable.

## 1 Introduction

Implant fracture is a possible complication in implant therapy, typically associated with late failures that are difficult to manage, usually involving the loss of both the implant and the prosthesis [1]. The incidence of implant fractures is relatively low [2, 3] and most frequently occurs around the abutment connection [2] and the implant body areas [3]. Three risk factors are considered major causes of implant fractures: defects in the design of the implant, non‐passive fit of the prosthetic structure, and biomechanical or physiological overload [4].

Narrow‐diameter implants (NDIs) are presumed to be at a higher risk of fracture, especially in the posterior region when associated with increased bone loss [5]. However, there is scarce information on the incidence of fractures of one‐piece NDIs used for overdenture retention. Since the stress concentration around the implant load is markedly lower in overdentures compared to fixed prostheses [6], it is unlikely that one‐piece implants would fracture throughout long‐term use.

**FIGURE 1:**
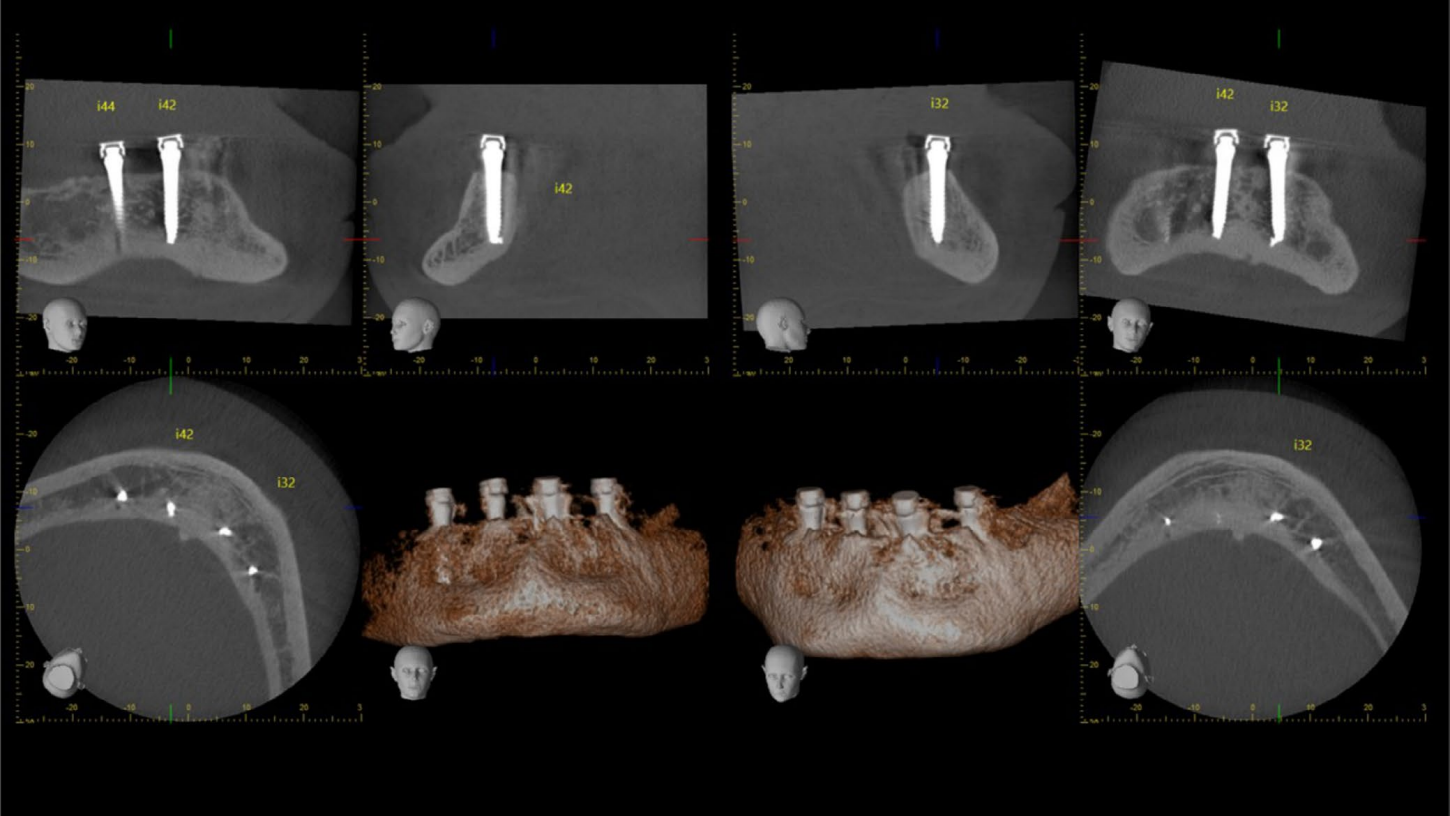
CBCT exam of a clinical case with apical implant fractures detected in post‐insertion assessments.

Nevertheless, a case of fracture of the implant tip was observed as an incidental finding during a follow‐up recall visit of a patient who received a four‐implant overdenture using one‐piece titanium–zirconium mini‐implants with miniaturized carbon‐based coated prosthetic connections for overdenture (Straumann Optiloc Mini Implant System, Institut Straumann AG, Basel, Switzerland). This case was observed in the clinical setting of the Department of Reconstructive Dentistry and Gerodontology of the University of Bern, Switzerland (Figure 1), and was considered as not related to the clinical use of the overdenture. Instead, it was hypothesized that the tapered design of the mini‐implant and its thin apical portion would be at risk of fracture under excessive torque forces during the surgical insertion of the implant.

Then, this unexpected occurrence was further included as a clinical/radiographic outcome in a subsequent clinical trial with a large sample (296 mini‐implants in 74 patients). This study aimed at testing a series of outcomes after treatment with a four‐mini‐implant mandibular overdenture using different surgical (flapped vs. flapless) and loading (immediate vs. delayed) protocols [7]. As a result, five apical implant tip fractures were identified in five patients (1.69% incidence), during surgery and/or in post‐operative radiographs, as reported previously [8]. Therefore, this prospective case series study aimed to describe the clinical and radiological features of implant tip fractures in further longitudinal investigations on this patient group. This study also aimed to identify potential risk factors associated with specific clinical and technical features of this one‐piece titanium–zirconium mini‐implant in order to minimize the occurrence of apical fractures.

## 2 Material and Methods

This case series describes clinical occurrences of apical tip fractures in one‐piece titanium–zirconium mini‐implants among patients treated with varying protocols for mandibular overdentures. The cases presented were derived from a randomized clinical trial (RCT) with a 2 × 2 factorial design that investigated surgical and loading protocols for mandibular overdenture rehabilitation using four one‐piece Ti–Zr mini‐implants per patient. The comprehensive clinical and radiographic outcomes of the 74 enrolled patients (296 implants) have been previously published [7]. The present manuscript had a specific focus on five patients (6.76% of the total sample) who experienced apical implant fractures, representing a nested case series within the broader RCT.

Patients included in the original clinical trial (*n* = 74) were assessed for the post‐insertion identification of apical fractures of one‐piece titanium–zirconium mini‐implants (Straumann Mini Implant System, Institut Straumann AG, Basel, Switzerland) used for mandibular overdenture treatment. The original study included all clinical trial participants testing different surgical and loading protocols for overdenture treatment using the four one‐piece mini‐implants. The original study protocol was prospectively registered in ClinicalTrials.gov (NCT04760457) in February 2021, and patient recruitment started in March 2021 (https://clinicaltrials.gov/study/NCT04760457).

The study complied with the standards of the Declaration of Helsinki concerning the ethical principles for medical research involving human subjects. Ethical approval by the local ethical committee was obtained within the context of the original study (CAAE 24833219.4.0000.5083). All the participants provided their informed consent before inclusion in the study.

Treatments were provided between April 2021 and January 2022 at the School of Dentistry of the Federal University of Goiás, Brazil. Previous reports on the short‐term surgical outcomes, drilling protocol, and implant primary stability, and peri‐implant outcomes have been published [7, 8]. This prospective case series report was prepared according to STROBE guidelines for reporting observational studies.

For all cases, the implant surgery was planned using a panoramic radiograph, cone‐beam computed tomography images (CBCT), as well as implant planning software (coDiagnostiX, Dental Wings Inc., Montreal, Canada). Criteria for the selection of implant position and length included bone availability and morphology, providing a minimum distance of 5 mm between implants, a minimum distance of 7 mm anteriorly to the mental foramen, with implants placed as parallel as possible and coincident with the path of insertion of the overdenture. Surgical access was randomized to either flapless or flapped surgery for all cases. All patients received four mini‐implants of 2.4 mm in diameter and 10, 12, or 14 mm in length (Straumann Mini Implants, Roxolid, SLA surface, Institut Straumann AG, Basel, Switzerland) inserted in the interforaminal area of the mandible. Implant site preparation was performed using a needle drill, followed by the 2.2 mm BLT Pilot Drill long. A minimum insertion torque of 35 N cm was required for immediate loading; the maximum insertion torque should not exceed 80 N cm as this may lead to implant damage. Final implant positioning was achieved with the handpiece at a maximum speed of 15 rpm or manually with the ratchet.

Baseline periapical radiographs were taken immediately after implant placement, and panoramic radiographs were obtained 6 weeks after surgery. When implant fractures were detected, an additional CBCT exam was performed. Follow‐up CBCT exams were required to monitor bone changes around the fractured apical tip of the mini‐implant and perforations of the cortical bone around the implant. In these cases, CBCT scans were acquired using an i‐CAT Precise Tomography System (Imaging Sciences International, Hatfield, PA, USA), with the protocol: 13 cm field of view, a voxel size of 0.25 mm, 120 kVp, 3.8 mA, and 30 s. The CBCT scans of the anterior mandible were taken with a limited field of view and standard protocols to minimize radiation exposure. The volume was exported in an axial multifile DICOM format, and the images were assessed qualitatively.

Clinical data concerning relevant surgical aspects, bone density assessment, bone anatomical features at the implant site, drilling protocol, and implant stability parameters were also recorded per the initial study protocol. The implant fractures were identified during surgical procedures for implant placement or in follow‐up radiographic images. The fractured implants were monitored up to the 3‐year follow‐up through periapical, panoramic radiographs, and CBCT exams to monitor bone changes around the implant fragment and to identify associated periapical pathologies. Clinical and radiographic exams were performed after 6 weeks and at the 3‐, 6‐, 12‐, 24‐, and 36‐month scheduled follow‐up visits for all patients from the original clinical trial, with a special focus on the implant fracture aspects for the incident patient group.

## 3 Results

A total of 74 edentulous patients (47 females; mean age 64.2 ± 7.5 years) were enrolled in the original randomized clinical trial (RCT) and received four one‐piece titanium–zirconium mini‐implants (Straumann Mini Implant System) placed in the interforaminal region of the mandible, resulting in a total of 296 implants. Ridge morphology was predominantly classified as Class III or IV according to the Cawood and Howell classification, and bone quality was mainly Type I or II based on the Lekholm and Zarb criteria, as assessed through preoperative CBCT imaging. Surgical procedures were evenly allocated between flapless and flapped approaches (37 patients in each group), and loading protocols were equally divided between immediate and delayed loading (37 patients each). Final insertion torque values ranged from 30 to 80 N cm, with the majority of implants (> 68%) achieving torque values above 45 N cm. The overall 1‐year implant survival rate was 100% [8], and no major biological or mechanical complications were observed during the subsequent follow‐up visits, extending up to 3 years after overdenture delivery.

Nevertheless, five clinical cases were identified in which apical fractures of the implant tip occurred. Three of these fractures were detected during the trans operative phase of implant placement. In all three cases, a high initial insertion torque (approximately 70 N cm) was recorded, followed by a loss of torque after the fracture occurred. This led to the repositioning of the affected mini‐implants to other implant sites, at which time the apical fractures became evident. The remaining two fractures were identified on periapical radiographs taken immediately after implant placement.

No further fractures were observed during the 3‐year follow‐up period. They were confirmed through routine annual periapical radiographs, which demonstrated the long‐term stability of all mini‐implants and confirmed that the fractures had occurred during the insertion phase rather than after functional loading. These cases were monitored throughout subsequent follow‐up visits, and their clinical and radiographic findings are presented below as a case series, alongside the randomized clinical trial outcomes.

The five patients with apical fractures of the mini‐implant were followed for 3 years; their main clinical and radiographic features were assessed and registered. Overall, all five implants remained clinically stable, functional, and asymptomatic throughout the 3‐year follow‐up. The main characteristics of each clinical case are detailed in Table 1.

**TABLE 1:** Main characteristics of the cases with the implant tip fractures.

Patient	Sex	Age (years)	Implant position	Implant length (mm)	Ridge form^a^	Bone type^b^	Final insertion torque (Ncm)	Detection	Flapless surgery
#1	Female	59	Central left	10	Well‐rounded	II	30	Transurgical	No
#2	Female	65	Lateral left	10	Knife‐edge	II	60	Follow‐up Rx	No
#3	Female	65	Lateral right	10	Flat	I	70	Transurgical	Yes
#4	Female	62	Central right	10	Well‐rounded	I	60	Post‐surgical Rx	No
#5	Male	64	Central right	12	Well‐rounded	II	40	Transurgical	Yes

^a^ According to Cadwood and Howell criteria.

^b^ According to Lekholm and Zarb criteria.

The five apical fractures (1.69% of all inserted implants) were identified either during implant insertion or on immediate postoperative radiographs. In all instances, only the apical tip of the implant fractured during placement. The implant body, prosthetic connection, and the osseointegration process remained unaffected. None of the implants with fractured tips were removed; all were retained in situ and continued to function effectively as part of the overdenture retention system. Follow‐up clinical assessments and radiographic evaluations revealed no evidence of marginal bone loss, peri‐implant inflammation, or implant mobility, indicating successful osseointegration in all five cases.

No specific pattern of implant position (central or lateral) was observed among the four implants inserted for each patient. Bone density of the implant sites was classified as Type I or II. Although the final implant insertion torque varied from 30 to 70 N cm, it was not possible to register the maximum torque achieved during the surgeries or even when the implant fracture actually occurred. Moreover, in two out of the five cases, the implant fracture was detected only in post‐insertion radiographs, while in the other three cases, it was detected during the surgery. Therefore, fractures were not associated with a specific clinical or anatomic feature. Moreover, individual descriptions of the clinical cases are presented in the following sessions.

### 3.1 Case #1 (Figure 2)

**FIGURE 2:**
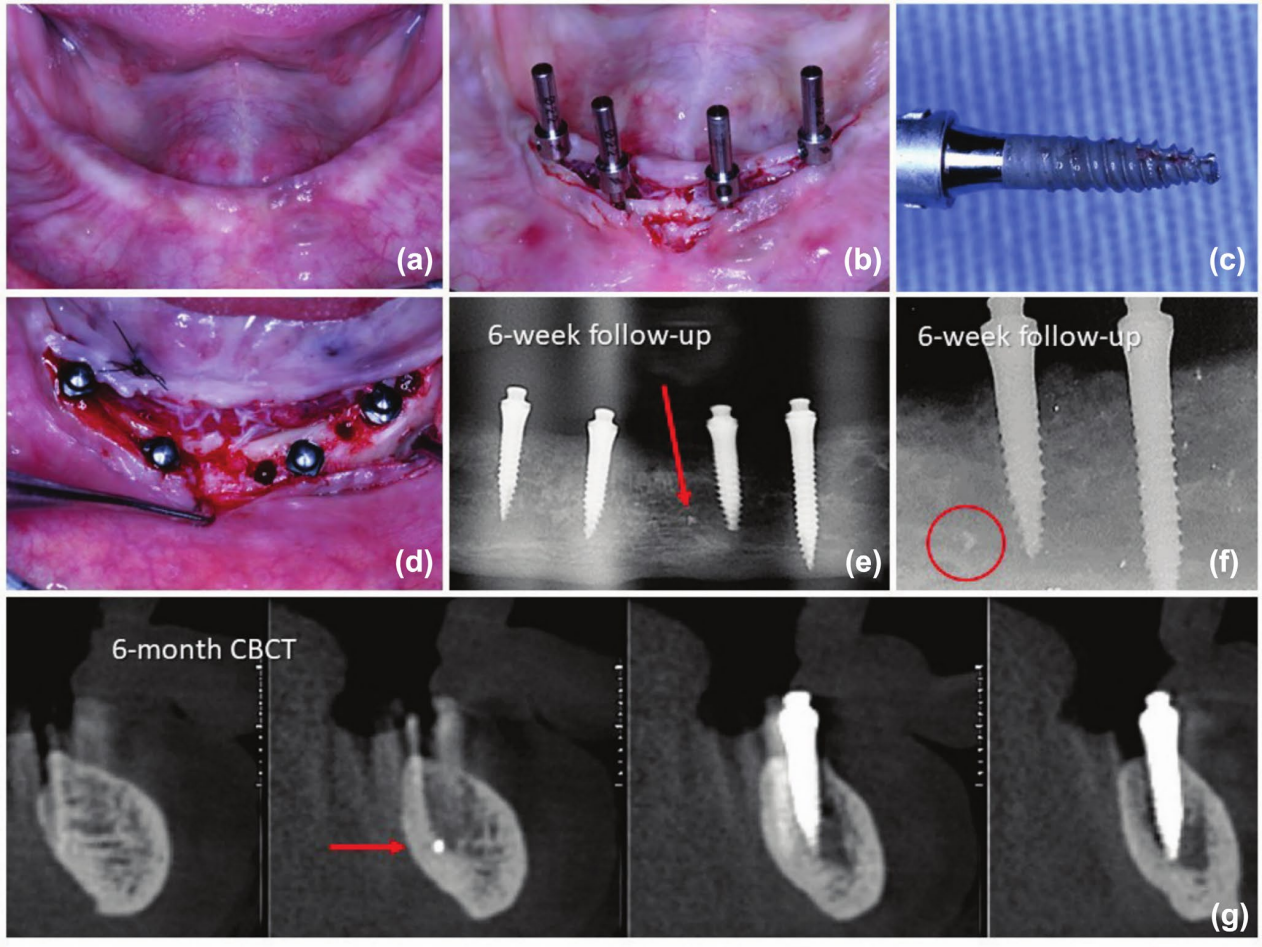
(a) Pre surgery view; (b) Initial transoperative view using a flapped approach; (c) Mini implant tip fracture; (d) Trans operative view showing the initial drilled sites and the final positions of the mini‐implants; (e) Panoramic radiograph at 6‐week follow‐up showing the fractured fragment; (f) Periapical radiograph of right implants at 6‐week follow‐up evidencing the fractured fragment; (g) CBCT at 6‐month follow‐up confirming the osseointegration of the implant and the fractured fragment.

A 59‐year‐old female patient received four mini‐implants in the mandible under a flapped and delayed loading protocol. The implant fracture occurred on the implant in the position of the left central incisor. The bone site presented with remaining bone volume classified as Class III according to Cawood and Howell and bone Type II according to Lekholm and Zarb (Figure 2a). The surgical procedure involved the placement of four implants using a flapped approach and a delayed loading protocol (Figure 2b,d). The initial implant site was prepared using only the needle drill, with lateral and vertical undersizing. During the implant placement in the central region on the left side, high torque values were achieved before reaching a very dense bone region and sudden torque decrease down to 5 N cm due to the rotation of the implant around its vertical axis, which led to the removal of the implant. At this point, the fracture of the tip of the mini‐implant was identified (Figure 2c). Following the surgical preparation guidelines, placing the same implant in a nearby bone site was decided. The final insertion torque achieved was 30 N cm. The panoramic and periapical radiograph taken 6 weeks after implant placement revealed the presence of the fractured fragment located anterior to the apex of the fractured implant and near the midline of the mandible (Figure 2e,f). The CBCT image scans at the 6‐month follow‐up confirmed the normal surrounding structures and proper osseointegration (Figure 2g). Longitudinal follow‐up during the 36 months also demonstrated satisfactory osseointegration for both the implant and the fractured fragment. There were no signs of bone loss or periapical pathology. The patient reported no discomfort or complications during the follow‐up, indicating a successful clinical outcome.

### 3.2 Case #2 (Figure 3)

**FIGURE 3:**
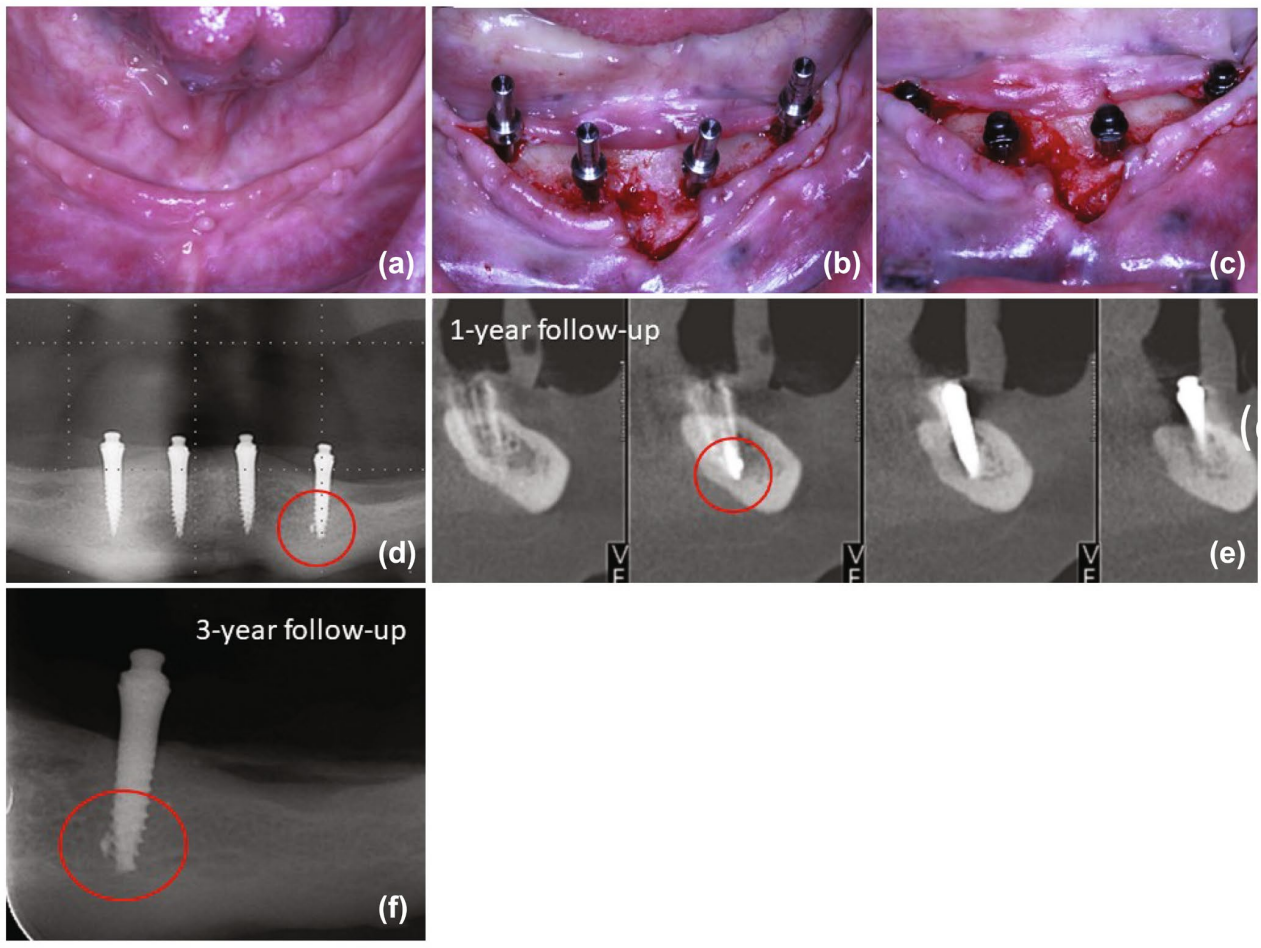
(a) Presurgical view; (b) Flapped surgery view after bone site preparation and paralleling posts in position; (c) Final aspect of the mini implant placement; (d) Panoramic radiograph at 6‐week follow‐up showing the fractured fragment close to the left lateral implant; (e) CBCT at 1‐year follow‐up showing the satisfactory osseointegration of the implant and the fractured fragment; (f) Periapical radiograph of the lateral left implant at the 3‐year follow‐up evidencing the absence of abnormalities.

A 65‐year‐old female patient underwent surgery for the placement of four one‐piece mini‐implants in the mandible, which exhibited significant vertical atrophy (Class VI, [9]) and bone Type II [10]. Another technical detail was the topography of the residual ridge, which had a sharp crest rising at the vestibular edge (Figure 3a). The surgical technique for implant placement was performed with a flapped approach and an immediate loading protocol (Figure 3b,c). Drilling was performed using the needle drill, followed by the BLT Pilot Drill 2.2 mm long. Due to this anatomical detail and the limited height, the central implants were placed more inferiorly to avoid fenestration of the mandibular base with drilling exceeding the available height limit, ensuring the implant did not surpass the inferior limit. Additionally, it was intended to prevent the elevated bony crest from interfering with the prosthetic adaptation. The final insertion torque of all implants was high (> 50 N cm). The fracture of the implant tip was identified in the panoramic radiograph taken at the 6‐week follow‐up visit (Figure 3d). Longitudinal follow‐up imaging demonstrated satisfactory osseointegration 3years post‐surgery of the implant and the fractured fragment, with no signs of bone loss or periapical pathology (Figure 3e,f).

### 3.3 Case #3 (Figure 4)

**FIGURE 4:**
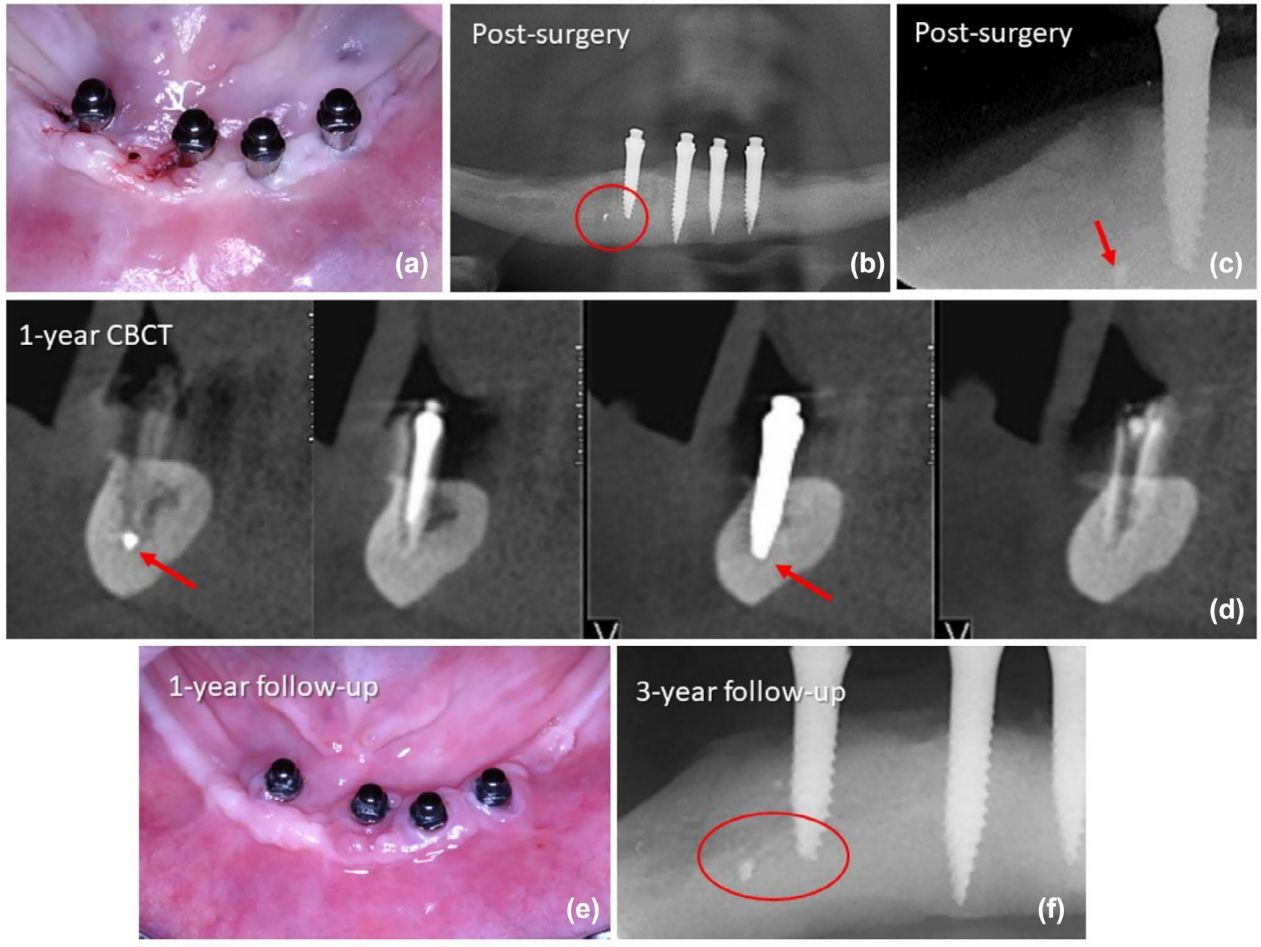
(a) Flapless transoperative view showing the first drilled site and the final position of the mini implants; (b) Post‐surgery panoramic radiograph showing a radiopaque image distal to the lateral right implant; (c) Post‐surgical periapical radiograph of mini implant and the radiolucent image of the first drilled site with the fractured fragment; (d) CBCT at the 1‐year follow‐up showing the satisfactory osseointegration of the implant and the fractured fragment. (e) Intra oral view at the 1‐year follow‐up; (f) Periapical radiograph of the right‐sided implants at the 3‐year follow up, showing the complete osseointegration around the implant and the fractured fragment.

A 65‐year‐old female patient with mandibular residual bone classified as Class VI according to Cawood and Howell and bone Type I as per Lekholm and Zarb. The surgical technique used was flapless and followed a delayed loading protocol (Figure 4a). The implant site was prepared with the needle drill and subsequently the 2.2 mm BLT Pilot Drill long was used. The patient had only the basal bone of the mandibular symphysis with high‐density bone tissue. Consequently, the depth of the preparations had to be carefully considered to avoid breaching and penetrating the base. The extent of drilling with the pilot drill was critical since its robust apex could potentially break through the bony floor of the surgical sockets. This procedure required precise control during drilling to maintain the integrity of the mandibular base. During the installation of the right lateral implant, rupture of the buccal bone plate was observed, and during the repositioning of this implant, a fracture of the implant tip was noted. Despite this complication, the decision was made to complete the insertion at another position, which was more mesially located. The implant was successfully placed, achieving a final insertion torque of 70 N cm, which ensured primary stability. A panoramic and a periapical radiograph were taken immediately after the surgery, which revealed the presence of the fractured fragment located in the posterior mandible (Figure 4b,c). Longitudinal follow‐up imaging and clinical evaluation, including periapical and CBCT, demonstrated satisfactory osseointegration and clinical performance at 1 year (Figure 4d,e). Both the implant and the fractured fragment showed no signs of bone loss or periapical pathology at the 3‐year follow‐up (Figure 4f). The patient reported no discomfort or complications during the follow‐up period, confirming the positive clinical outcome of the procedure.

### 3.4 Case #4 (Figure 5)

**FIGURE 5:**
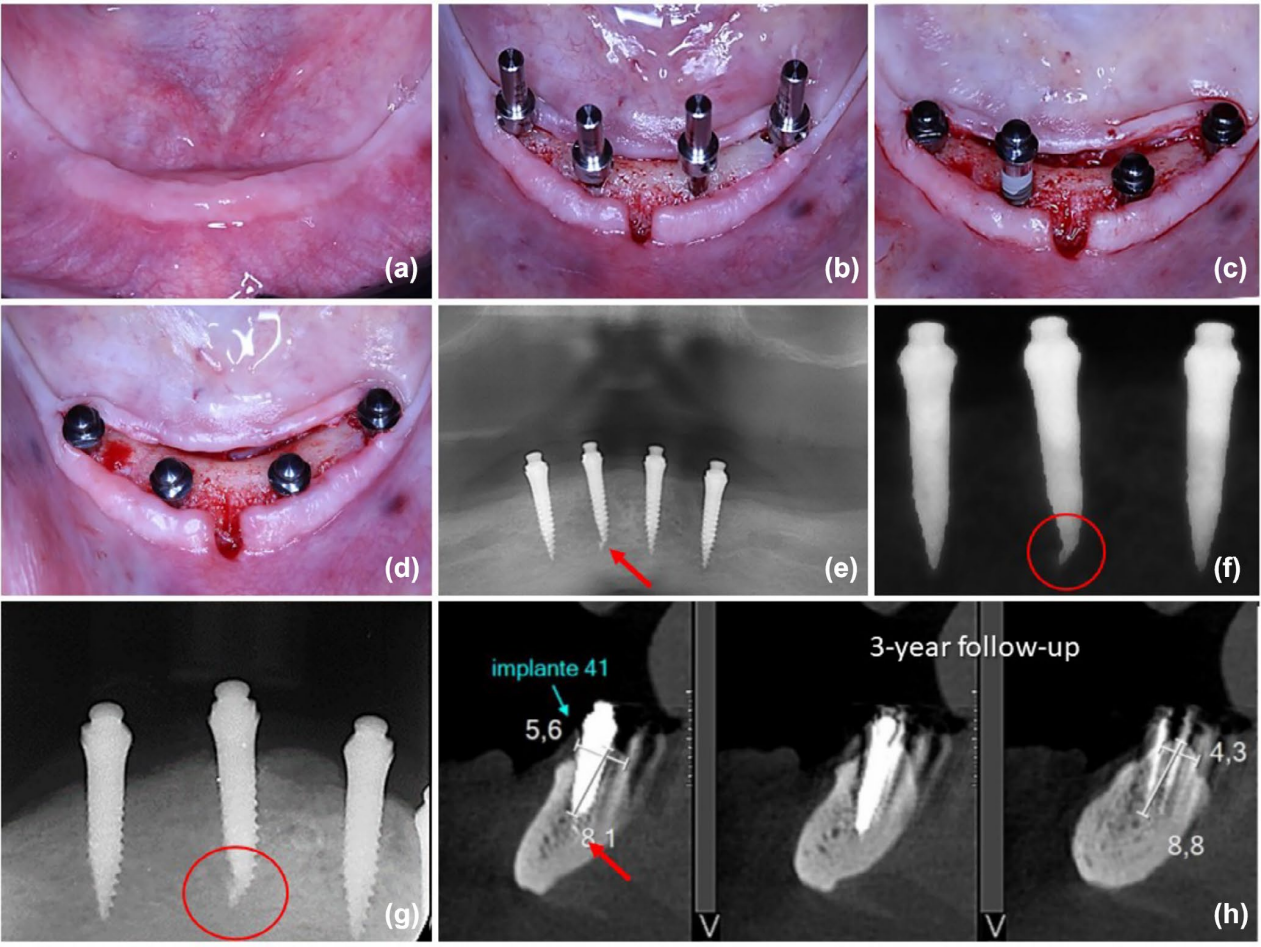
(a) Presurgical view of the edentulous mandible ridge; (b) Transoperative view of flapped surgery; (c) Insertion of mini implant in the central right position; (d) Intraoral aspect of the placed mini implants; (e) Panoramic radiograph at 6‐week follow‐up evidencing a radiopaque image suggestive of mini implant tip fracture; (f) 3D reconstruction of CBCT showing the position of the fractured fragment using a boneless filter; (g) Periapical radiograph at 2‐year follow‐up of the central right implant evidencing the fractured implant tip; (h) CBCT image scans at the 3‐year follow‐up, showing the fractured fragment located in a dense cortical bone and revealing satisfactory osseointegration.

A 62‐year‐old female patient was evaluated for implant overdenture treatment in the mandible. The patient exhibited mandibular residual bone classified as Class III according to Cawood and Howell, indicating significant bone resorption with an adequate but compromised ridge, and bone Type I according to Lekholm and Zarb, denoting dense cortical bone (Figure 5a). The surgical technique for implant placement followed a flapped protocol. A median vertical incision was chosen to facilitate lateral displacement and ensure the preservation of the mental nerves, which had become superficial on the crest of the ridge due to advanced bone resorption (Figure 5b,c). Drilling was performed using the needle drill, followed by the 2.2 mm BLT Pilot Drill long. The high density of the bone allowed for the achievement of high final insertion torques (> 50 N cm). The mandibular complete denture was relieved in the regions of the mini‐implants, and delayed loading was carried out after 6 weeks. A panoramic radiograph taken at the 6‐week follow‐up revealed a fracture at the tip of the right central implant (Figure 5d). Longitudinal follow‐up imaging demonstrated satisfactory osseointegration of the implant and the fractured fragment, with no signs of bone loss to 36 months post‐surgery (Figures 5e–h). The patient reported no discomfort or complications during the follow‐up, indicating a successful clinical outcome.

### 3.5 Case #5 (Figure 6)

**FIGURE 6:**
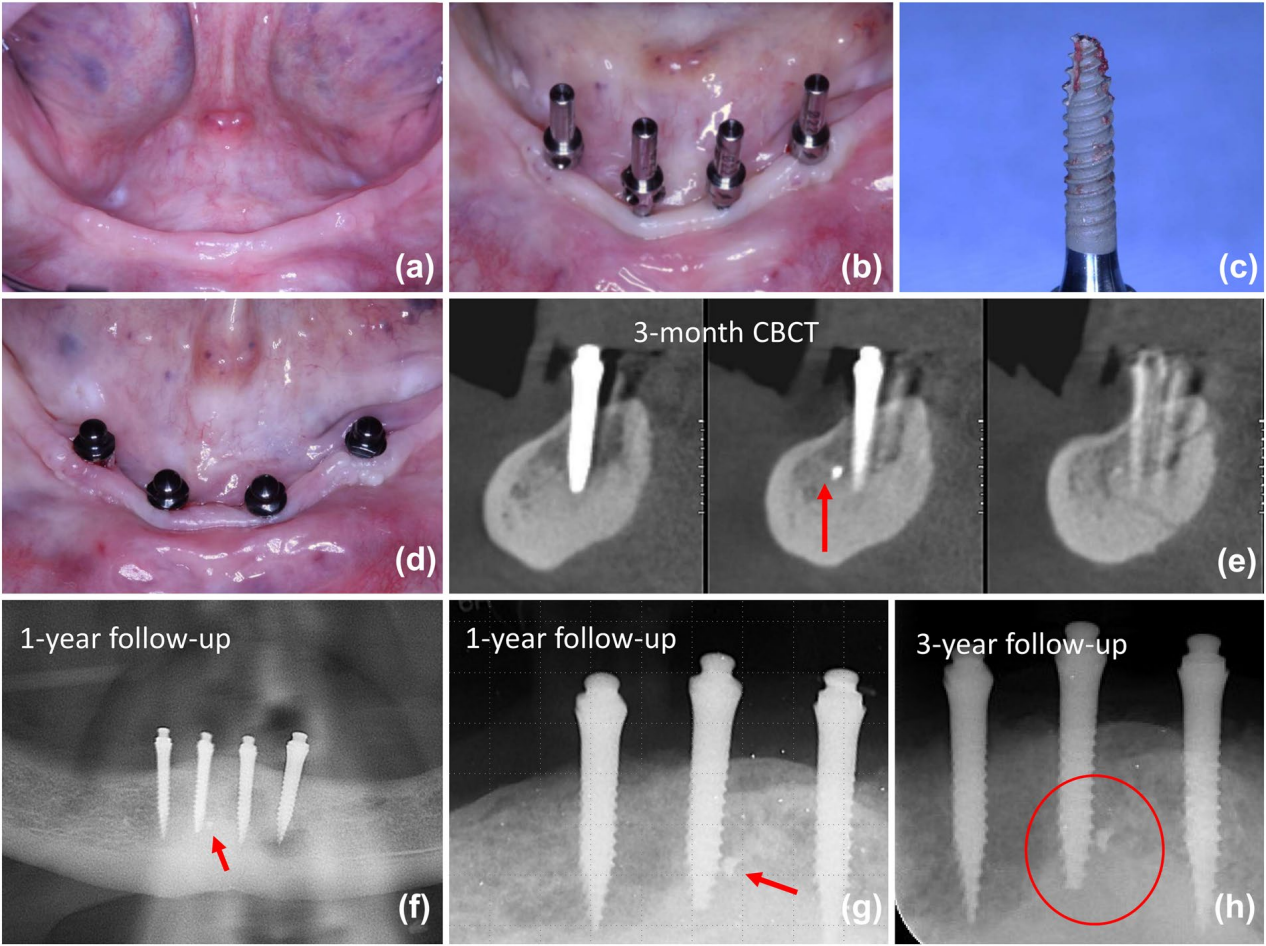
(a) Pre surgery mandibular view; (b) Flapless transoperative view after bone preparation and the paralleling posts in position; (c) Mini implant with apical fracture; (d) Immediate post‐surgery view; (e) CBCT image scans at the 3‐month follow up showing a satisfactory osteointegration and the fractured fragment located in a dense cortical bone region; (f) Panoramic radiograph at the 1‐year follow‐up; (g) Periapical radiograph of the central right implant at the 1‐year follow up, evidencing the osseointegration of the fractured fragment (h) Periapical radiograph at the 3‐year follow up, with no signs of bone alterations.

A 64‐year‐old male patient presented with mandibular residual bone classified as Class III according to Cawood and Howell and bone Type II as per Lekholm and Zarb. The patient exhibited moderate bone resorption with adequate ridge height but compromised density, especially in the superficial layers (Figure 6a). The surgical placement of four mini‐implants in the mandible was performed flapless (Figure 6b). During the drilling process, it was noted that the superficial bone at the maximum drilling height had low density, whereas the basal bone was extremely dense. This posed a challenge in achieving optimal primary stability for the implants; sub‐drilling to a depth of approximately 1.5 mm was performed to address this. However, this depth was insufficient for the final placement of the central implants. As a result, the implants were removed, and deeper drilling was carried out with a needle drill into the dense basal bone, allowing the final placement of the implants. During the placement of the right central implant, a fracture of the implant tip was observed upon removal (Figure 6c). Despite this complication, it was decided to proceed with the mini‐implant placement in the same site, which achieved a final insertion torque of 40 N cm, ensuring sufficient primary stability (Figure 6d). Delayed loading of the mandibular complete denture was implemented after 6 weeks. The 3‐month follow‐up imaging was obtained to monitor the osseointegration process (Figure 6e). The images demonstrated satisfactory osseointegration of the implant and the fractured fragment, with no signs of bone loss or periapical pathology 1 year (Figure 6f,g) and 3years post‐treatment (Figure 6h). The patient reported no discomfort or complications throughout the follow‐up period, indicating a successful clinical outcome.

## 4 Discussion

This case series highlights the occurrence of implant tip fractures during the insertion of one‐piece titanium–zirconium mini‐implants for mandibular overdentures, providing valuable insights and considerations for clinical practice. Based on post‐insertion CBCTs, it was hypothesized that implant tip fracture is an uncommon complication but with no negative impact on treatment prognosis. The fractured tips did not compromise the overall stability and osseointegration of the implants, as evidenced by longitudinal follow‐up imaging that showed satisfactory osseointegration 36 months post‐surgery, with no signs of bone loss, periapical pathology, or patient discomfort. This suggests that the fracture of the implant tip may not necessarily affect the long‐term success of the treatment, and implant retrieval may not be needed.

These findings reinforce that an apical tip fracture should not be considered a true implant failure. Rather, it may represent a mechanical event which, although infrequent, does not compromise implant function or long‐term outcomes when the remaining portion of the implant remains stable and properly osseointegrated. The implant's specific design and material characteristics, namely, its narrow diameter (2.4 mm), one‐piece configuration, and titanium–zirconium alloy, may influence its behavior under high insertion torque, particularly in dense cortical bone. Further mechanical testing and long‐term clinical studies are needed to elucidate the structural limits of such implant systems and to establish evidence‐based clinical guidelines for torque management during insertion in high‐density bone sites.

The mini‐implant system used in this study is made from the titanium–zirconium material, commercially known as Roxolid (Institut Straumann AG, Basel, Switzerland), which aimed to provide high strength and load resistance of implants with small diameter that is higher than the standard titanium implants [11]. Therefore, it seems unlikely that the apical fracture is related to poor properties of the implant material and design. Conversely, the incidence of implant tip fractures appears to be associated with dense cortical bone and high insertion torque values. In the reported cases, dense basal bone and inclined cortical bone required high insertion torques, sometimes reaching up to 80 N cm, which likely contributed to the fractures. This observation is consistent with the findings of Gealh et al. [4], who identified high insertion torque and dense bone conditions as risk factors for implant fracture.

The radiographic and CBCT images illustrate specific anatomical aspects (hard basal cortical bone with an inclined cortical surface in the lingual aspect of the alveolar bone) that could explain the occurrence of fractures. The images highlight the dense cortical bone and the anatomical challenges faced during implant placement, reinforcing the need for careful planning and execution in such cases. The use of the Straumann Optiloc Mini Implant System, with its one‐piece titanium–zirconium design, was critical in these cases. The surgical technique varied between flapless and flapped approaches, and both immediate and delayed loading protocols were employed. The choice of technique and protocol was tailored to each patient's anatomical and clinical conditions, demonstrating the flexibility and adaptability required in implant therapy.

A previous study focused on the drilling protocols in this patient cohort [12] reported that site‐specific preparation would be needed to achieve appropriate final insertion torque, especially for immediate loading, while special care should be taken for hard‐density bone sites to minimize the risk of implant and instrument fracture due to excess torque values greater than 65 N cm. In addition, since the surgical protocols for mini‐implants would demand simplified procedures and instruments for implant bed site preparation and insertion, achieving satisfactory primary stability may be challenging, especially when immediate loading and conservative surgical access are planned [12].

The observed incidence rate of implant tip fractures in this case series was 1.69% (five fractures out of 296 mini‐implants in 74 patients) [8]. This relatively low incidence rate suggests that while implant tip fractures are a notable complication, they are not exceedingly common in clinical practice. Previous studies have also reported low incidence rates for implant fractures, further underscoring the uncommon nature of this complication [2, 3].

Another point that may be discussed is how to react when an implant tip fracture occurs mid‐surgery, such as occurred in Cases #1 and #3. In Case #3, the osteotomy was prepared, and the implant was placed; during implant insertion, the buccal plate fractured, and the implant was removed. It was then that the surgeon noted that the implant tip was fractured. A new osteotomy was prepared in the neighboring mesial site, and it was decided to insert the same implant to simulate a situation where no other implant was available and to observe any complications at follow‐up. Moreover, no attempt to retrieve the implant tip fragment was performed since it may not be feasible without extensive bone removal to access the region of the fracture. At the 3‐year follow‐up, no complications were reported despite the implant tip fracture, suggesting that there is no need to replace the fractured mini‐implant since satisfactory stability and long‐term survival may be achieved even with a fractured apical tip.

It is important to highlight that the inclusion of other suitable treatment options was not explored in this study; rather, the primary objective was to evaluate whether treatment could be successfully achieved using a combination of flapless surgery and immediate loading, an approach confirmed as feasible in the clinical trial. The indication for using four one‐piece titanium–zirconium mini‐implants (diameter: 2.4 mm) was based on a combination of clinical, anatomical, and prosthetic considerations, and aligned with the inclusion criteria of the randomized clinical trial from which this case series was derived. Several patients presented with moderate to advanced mandibular ridge resorption, featuring knife‐edge or well‐rounded residual ridges (Cawood and Howell Class III to VI), and alveolar widths insufficient to accommodate two regular‐diameter implants (≥ 3.5 mm) without the need for additional surgical interventions such as bone grafting. In this context, narrow‐diameter one‐piece implants provided a minimally invasive, flapless treatment alternative, particularly advantageous for elderly or medically compromised patients. Moreover, the trial aimed to assess the clinical outcomes of a simplified, cost‐effective solution for mandibular overdenture support, suitable for public healthcare systems and resource‐limited settings, given that it may be less costly than standard implants placed using conventional surgical and loading protocols. Functional loading was performed with caution, following a standardized protocol using solitary resilient attachments (Optiloc), which may have contributed to favorable load distribution and reduced mechanical stress on the implants. All fractures reported in this study were exclusively associated with the surgical insertion phase, with no evidence of fracture occurring during functional loading over the 3‐year follow‐up period.

This case series provides valuable insights into the clinical scenarios, which may lead to implant apical fractures. Each case emphasized different aspects of bone density, anatomical challenges, and surgical techniques, contributing to a comprehensive understanding of this complication. For instance, one case involved the placement of implants in a patient with high‐density basal bone, which required careful depth consideration to avoid breaching the bone floor. The analysis of these risk factors indicates that high insertion torque in dense bone conditions would highly contribute to implant tip fractures. The findings underscore the importance of meticulous surgical planning and execution, particularly in patients with high bone density. Adjusting drilling protocols and insertion techniques to accommodate the bone's physical properties can mitigate the risk of fractures and enhance clinical outcomes. Continued research and clinical vigilance are essential to further minimize the risk of such complications and optimize patient care.

## 5 Conclusion

The findings from this 3‐year prospective case series of implant tip fracture in interforaminal mini‐implants allow us to draw the following conclusions:

- Implant tip fracture is an uncommon finding that does not result in any biological or technical complications after a 3‐year follow‐up.
- Risk factors associated with implant tip fracture include site anatomy, bone density, and quality as well as implant insertion torques.
- Findings suggest that an increased risk of fracture was associated with high insertion torques (around 80 N cm) and implant insertion in areas where the tip engages into a hard basal cortical bone with an inclined cortical surface in the lingual aspect of the alveolar bone.
- The fracture of the implant tip may not necessarily affect the long‐term success of the treatment; implant retrieval may not be needed.
- The successful management of these cases, with satisfactory mid‐term outcomes, highlights the resilience of modern implant systems and the importance of tailored surgical approaches.

## Author Contributions

**Cláudio Rodrigues Leles:** conceptualization, methodology, statistics, original draft writing, review and editing, funding acquisition, project administration. **Thalita Fernandes Fleury Curado:** data collection and investigation, data analysis and interpretation, original draft preparation, project administration. **José Luiz Rodrigues Leles:** conceptualization, methodology, investigation, draft writing, review and editing. **Nádia Lago Costa:** data analysis and interpretation, original draft writing, review and editing. **Manrique Fonseca:** data analysis and interpretation, original draft writing, review and editing. **Martin Schimmel:** data analysis and interpretation, original draft writing, review and editing, funding acquisition. **Gerald McKenna:** data analysis and interpretation, original draft writing, review and editing, funding acquisition.

## Conflicts of Interest

Cláudio R. Leles, Manrique Fonseca, Martin Schimmel, and Gerald McKenna are the recipients of other funding from Institut Straumann AG and the ITI. The other authors do not report any conflicts of interest related to the present study.

## Data Availability

The data that support the findings of this study are available from the corresponding author upon reasonable request.
